# Constitutive modeling using structural information on collagen fiber direction and dispersion in human superficial femoral artery specimens of different ages

**DOI:** 10.1016/j.actbio.2020.11.046

**Published:** 2020-12-03

**Authors:** Majid Jadidi, Selda Sherifova, Gerhard Sommer, Alexey Kamenskiy, Gerhard A. Holzapfel

**Affiliations:** a Department of Mechanical and Materials Engineering, University of Nebraska-Lincoln, Lincoln, NE, USA; b Institute of Biomechanics, Graz University of Technology, Stremayrgasse 16-II, Graz 8010, Austria; c Department of Biomechanics, Biomechanics Research Building, 6160 University Drive South, University of Nebraska Omaha, Omaha, NE 68182, USA; d Department of Structural Engineering, Norwegian University of Science and Technology (NTNU), Trondheim, Norway

**Keywords:** Superficial femoral artery, Constitutive model, Collagen structure, Second-harmonic generation imaging, Two-photon fluorescence imaging, Biaxial data

## Abstract

Arterial mechanics plays an important role in vascular pathophysiology and repair, and advanced imaging can inform constitutive models of vascular behavior. We have measured the mechanical properties of 14 human superficial femoral arteries (SFAs) (age 12–70, mean 48±19 years) using planar biaxial extension, and determined the preferred collagen fiber direction and dispersion using multiphoton microscopy. The collagen fiber direction and dispersion were evaluated using second-harmonic generation imaging and modeled using bivariate von Mises distributions. The microstructures of elastin and collagen were assessed using two-photon fluorescence imaging and conventional bidirectional histology. The mechanical and structural data were used to describe the SFA mechanical behavior using two- and four-fiber family invariant-based constitutive models. Older SFAs were stiffer and mechanically more nonlinear than younger specimens. In the adventitia, collagen fibers were undulated and diagonally-oriented, while in the media, they were straight and circumferentially-oriented. The media was rich in collagen that surrounded the circumferentially-oriented smooth muscle cells, and the elastin was present primarily in the internal and external elastic laminae. Older SFAs had a more circumferential collagen fiber alignment, a decreased circumferential-radial fiber dispersion, but the same circumferential-longitudinal fiber dispersion as younger specimens. Both the two- and the four-fiber family constitutive models were able to capture the experimental data, and the fits were better for the four-fiber family formulation. Our data provide additional details on the SFA intramural structure and inform structurally-based constitutive models.

## 1. Introduction

Arterial mechanics plays an important role in vascular physiology and pathophysiology, and it profoundly influences the design of devices and materials for open and endovascular repair [1-4]. Extension-inflation and planar biaxial extension tests are frequently used to characterize the multiaxial stress-stretch responses of arteries [5-12] and to obtain sufficient data for mathematical modeling of their mechanical behavior [13-18]. The two main types of constitutive models that are frequently employed to capture arterial mechanical characteristics are structural and phenomenological. Structural models [18-28] describe the behavior of tissue by modeling individual responses of its constituents and their interactions. They are instrumental for exploring various mechanobiological mechanisms but require detailed information on the organization, properties, loading conditions, and contact characteristics of each constituent at each deformation state, which, for many cases, is difficult, if not impossible, to measure directly. Conversely, purely phenomenological models [29-33] are descriptors of stress-stretch responses, and have the advantage of a straightforward computational implementation, but they provide no insights into tissue structure and function. A hybrid type of constitutive models are structurally-motivated phenomenological formulations [34-39]. They offer the benefits of relative simplicity and robustness to limited experimental data, while also having the potential to become more structurally-based when appropriate information is available. With the advances in imaging techniques, this new type of models has become increasingly popular as the investigators were able to explicitly include preferred fiber orientation, dispersion, or active cellular responses into their constitutive formulations [14,37,40-45].

Although basic structural characteristics that can inform hybrid constitutive models are measurable through histology or simple imaging modalities, these data are usually limited by their 2D nature that is often insufficient to characterize the out-of-plane arterial constituents. More advanced imaging modalities, such as multiphoton microscopy [46-48], have recently been utilized to characterize the complex anisotropic structure of the arterial wall. Structural data for the aorta [46,49-53], coronary arteries [54], and carotid arteries [55-58] have been reported, but much less attention has been given to the arteries of the lower limb. These arteries undergo severe mechanical deformations during limb flexion [59-61] and have a distinct structure with longitudinally-oriented elastic fibers in the external elastic lamina that facilitate arterial deformations during locomotion [5,62-64]. Arteries of the lower limb are known to develop atherosclerotic occlusive disease (peripheral arterial disease, PAD) that has one of the worst clinical outcomes of all arterial beds [65-70]. Characterization of the mechanical properties, the structure and the function of these arteries can help better understand the reasons for these poor clinical outcomes, and help to develop more efficient therapies for PAD.

The goal of the present study was to characterize the microstructure and the *ex vivo* biaxial mechanical properties of human superficial femoral arteries (SFA) within a wide range of age. The preferred direction of the collagen fibers and their dispersion around this direction were determined using multiphoton microscopy (MPM) with second-harmonic generation (SHG) imaging. These data were then used to inform structurally-motivated constitutive models of the SFA behavior which can be used for more advanced computational modeling.

## 2. Methods

### 2.1. Planar biaxial extension testing

Proximal SFAs (approximately 1–2 cm distal to the profunda femoris artery) from 14 subjects (age range 12–70 years, mean age 48±19, 43% male) were procured by Live On Nebraska within 24h of death after obtaining the informed consent from next of kin. Subject demographics are summarized in Table 1. Although these data were not explicitly used in the model, they provide additional information about the evaluated specimens for future studies, and are complementary to the structural and mechanical information presented below. Planar biaxial extension tests of fresh tissues were performed using a CellScale Biotester (CellScale, Waterloo, ON, Canada) equipped with 2.5N load cells. Specimens with dimension 13 × 13mm (when permitted by arterial diameter) were submerged in 0.9% phosphate-buffered saline (PBS) at 37°C, and the arterial directions were aligned with the test axes. Tissues were preconditioned using 20 cycles of loading and unloading to achieve a repeatable response. After preconditioning, 21 multi-ratio stretch-controlled protocols at 0.01 s^−1^ strain rate were executed to obtain sufficient data density for the constitutive modeling. These protocols ranged from circumferential:longitudinal stretch ratios of 1:0.1 to 1:0.9 and 0.9:1 to 0.1:1 with a 0.1 step, intermixed with three 1:1 equibiaxial protocols at the beginning, middle, and the end of the test sequence to ensure that the specimens did not accumulate damage. The maximum stretch level in the circumferential and longitudinal directions was selected for each specimen to ensure nonlinearity in the stress-stretch response while avoiding excessive stretch that can cause tissue damage. This was achieved by equibiaxially stretching the samples to 0.7-1.2N prior to preconditioning and estimating the maximum stretch in each direction. The deformation gradient was then measured using a top-mounted camera by tracking the movements of graphite markers, and the experimental Cauchy stresses were computed for both directions. For details regarding the mechanical testing, the reader is referred to Kamenskiy et al. [62] and Jadidi et al. [5].

### 2.2. Histological analysis

Histological analysis was performed on transverse sections and 13mm longitudinal strips obtained from the arterial segments immediately adjacent to the mechanically tested specimens. All tissues were fixed in methacarn, dehydrated in 70% ethanol, embedded in paraffin, sectioned using a microtome, and stained with Verhoeff-Van Gieson (VVG) stain to visualize elastin, and with Masson’s Trichrome (MTC) stain to visualize collagen and cell cytoplasm.

### 2.3. Multiphoton microscopy

#### 2.3.1. Sample preparation

Tubular formalin-fixed specimens were shipped to the Institute of Biomechanics at Graz University of Technology in Austria to perform structural analyses. The analyses were carried out on two formalin-fixed rectangular 5 × 4mm (circumferential × longitudinal, in-plane imaging) and 2 × 4mm (circumferential × radial, out-of-plane imaging) pieces adjacent to the biaxially-tested specimens. Arteries were optically cleared using a graded ethanol and benzyl alcohol benzyl benzoate (BABB) series [53]. This included first washing them with PBS to remove any paraformaldehyde residue, dehydrating the tissues in ethanol series consisting of 50, 70, 95, and 2 × 100% ethanol solutions (each for ~45min), and then submerging the specimens into a BABB:ethanol solution (1:1) for 4h before placing them in 100% BABB for at least 12h prior to imaging.

#### 2.3.2. Imaging

MPM imaging was performed at the Core Facility Bioimaging of the Biomedical Center at Ludwig Maximilian University of Munich in Germany using an upright Leica SP8 multiphoton microscope equipped with a pulsed InSight DS+ laser and a Leica HC FLUO-TAR 16 × /0.60 IMM BABB immersion objective with a working distance of 2.5mm. To avoid reflections, the samples and the lens of the objective were fully immersed in BABB during imaging using BABB proof containers. In-plane image stacks were acquired from the circumferential-longitudinal plane through the specimen thickness with a sampling of 690 × 690 × 5μm (image-size × step-size, 1024 × 1024px), and the out-of-plane images were obtained from the circumferential-radial plane with a sampling of 690 × 690μm (image-size, ~2300 × 2300px). The excitation wavelength of the MPM laser was tuned to 880nm. An SP 680 blocking filter was installed to block the excitation light with wavelengths greater than 680nm from reaching the detectors, and a beam splitter (BS 488LPXR) to split the light at the wavelength of 488nm. An internal conventional photomultiplier tube detector was used to obtain the auto-fluorescence signal from the elastin in the 491–561nm wavelength range in the two-photon excitation fluorescence (TPF) mode. Simultaneously, SHG signal of fibrillar collagen type I was collected in the forward direction by an external photomultiplier tube detector using a 420/40 bandpass filter. Note that the endogenous SHG signal in the artery arises primarily from the fibrillar type I collagen [46,71].

#### 2.3.3. Structural parameters

SHG images were used to obtain the structural parameters according to their definitions documented in Holzapfel et al. [36]. In particular, the mean fiber angle *α*, the in-plane dispersion parameter *κ*_ip_, and the out-of-plane dispersion parameter *κ*_op_ were obtained by averaging the in-plane image stacks and the out-of-plane images through-the-thickness. This was achieved in three steps: image processing, angle data extraction, and data fitting.

**Fig. 1.**
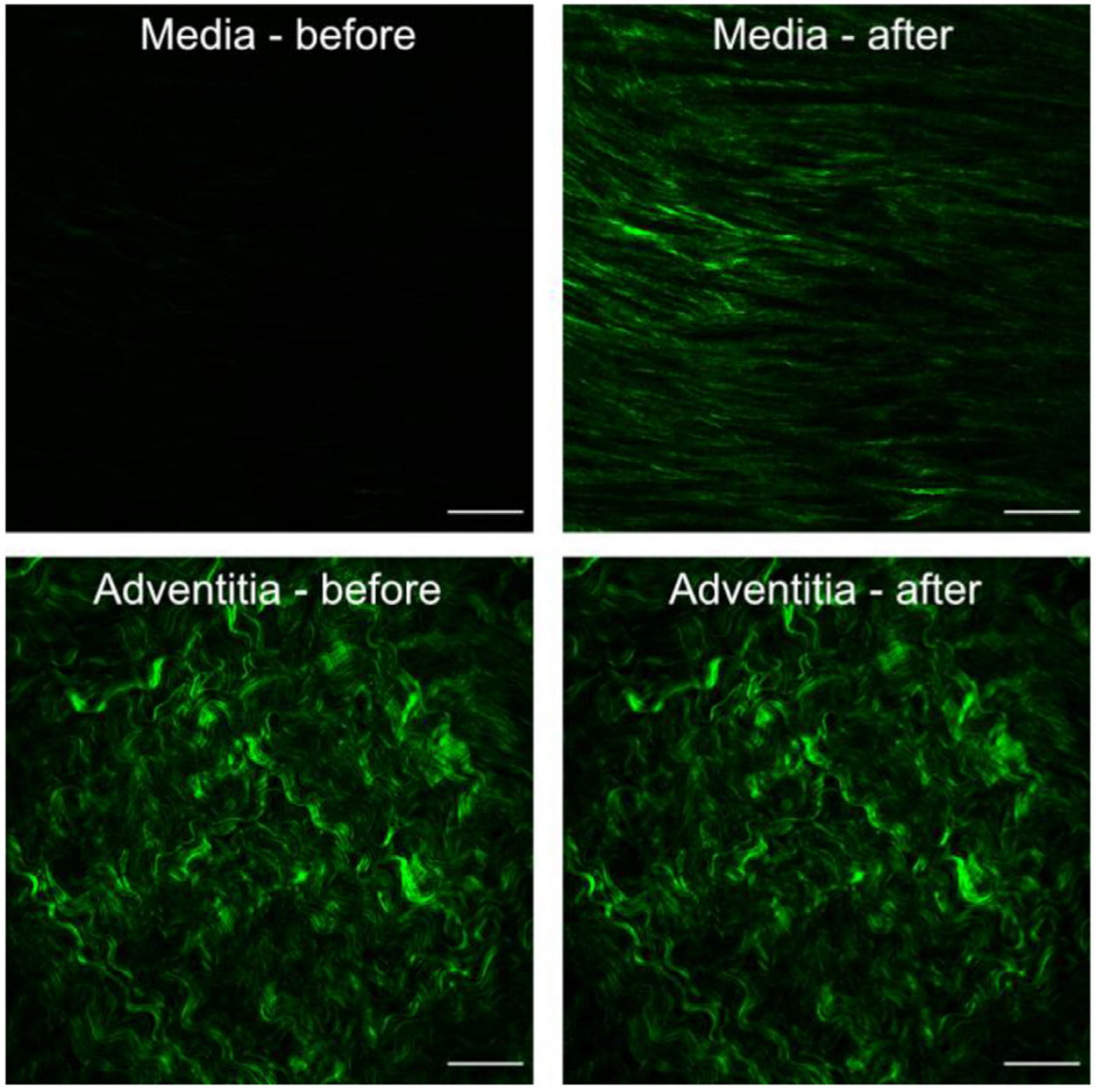
The effect of performing contrast enhancement and noise despeckling on the medial (top) and adventitial (bottom) images prior to collagen fiber angle measurement. Scale bar: 100*μ*m.

Since the SHG signal from the adventitia was very strong compared with the intima and media prior to the collagen fiber angle measurements, contrast enhancement and noise despeckling were applied on all stacks (see Fig. 1). By using a custom-written MATLAB code, we have removed the maximum intensities if white noise was present, e.g., due to calcification or dust particles, and applied median filtering in two steps, and image normalization after the first median filtering. Subsequently, discrete angular distributions of relative amplitudes of collagen fibers, resembling the fiber orientation [59], were extracted from all images by combining Fourier power spectrum analysis and wedge filtering [53] using a wedge width of 5°.

**Fig. 2.**
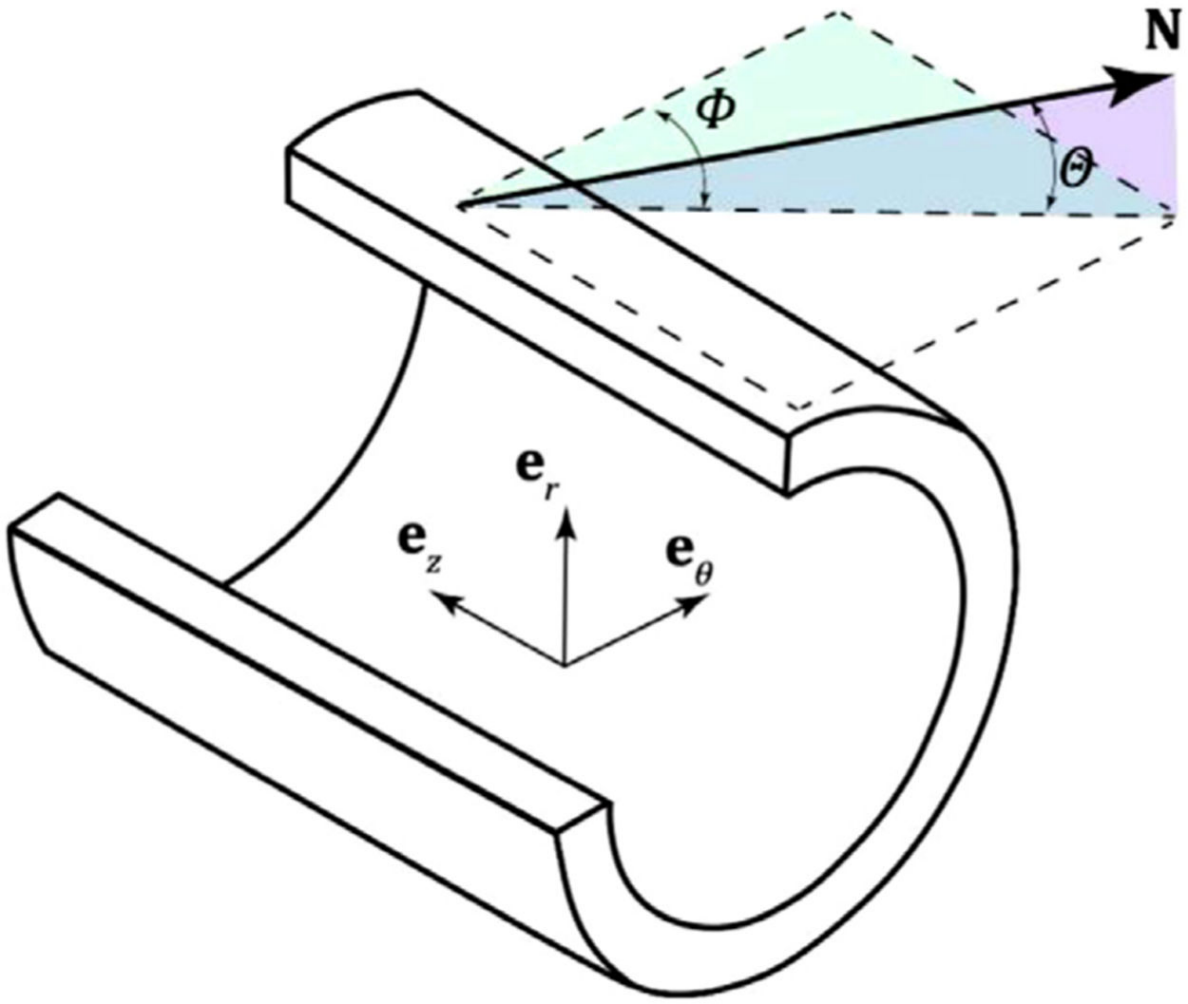
Unit vector **N** representing an arbitrary fiber direction in the stress-free state, defined by the angles Θ (angle between **N** and the circumferential-longitudinal plane) and Φ (angle between the projection of **N** in the circumferential-longitudinal plane and the circumferential direction).

The extracted fiber angle data were then used to obtain the mean fiber angle, and the in-plane and out-of-plane dispersions [36]. Briefly, the unit vector **N**, representing an arbitrary fiber direction in the stress-free state, was described in a rectangular Cartesian coordinate system with the basis vectors **e**_*θ*_, **e**_*z*_ and **e**_*r*_, corresponding to the circumferential, longitudinal, and radial directions, respectively (see Fig. 2), and **N** is given by

(1) $$N(\Phi ,\Theta )=\cos\;\Theta \;\cos\;\Phi e_{\theta }+\cos\;\Theta \;\sin\;\Phi e_{z}+\sin\;\Theta e_{r}\;,$$

where Φ ∈ [0 , 2*π*] denotes the in-plane, and Θ ∈ [−*π*/2 , *π*/2] the out-of-plane angle. The probability density function *ρ*(**N**) describing the distribution of the fiber orientation **N** is given as the multiplication of independent in-plane and out-of-plane distributions [36], i.e.

(2) $$\rho (N)=\rho (\Phi ,\Theta )=\rho_{\text{ip}}(\Phi )\rho_{\text{op}}(\Theta )\;,$$

where *ρ*_ip_ (Φ) and *ρ*_op_(Θ) are given by particular choices of von Mises distributions as

(3) $$\rho_{\text{ip}}(\Phi )=\frac{\text{exp}\;[a\;\cos2(\Phi \pm \alpha )]}{I_{0}(a)}\;,$$

(4) $$\rho_{\text{op}}(\Theta )=2\sqrt{\frac{2b}{\pi }}\frac{\text{exp}\;[b(\cos\;2\Theta -1)]}{\text{erf}(\sqrt{2b})}\;.$$

**Fig. 3.**
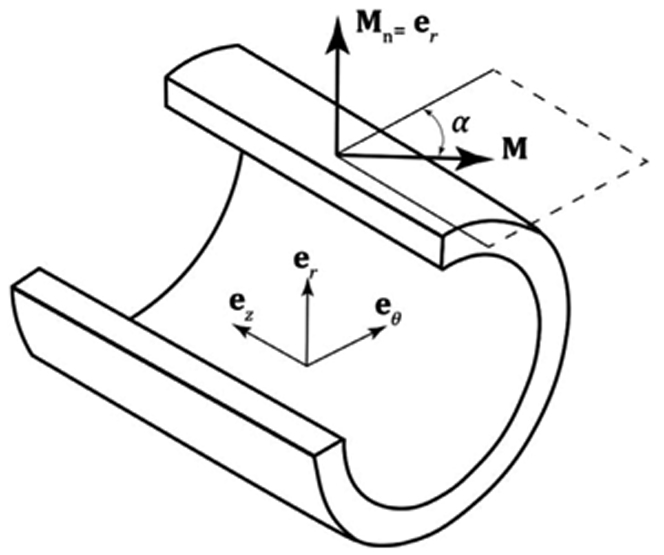
Unit vector **M** representing the mean fiber direction in the circumferential-longitudinal plane of the stress-free artery. Here *α* is the angle between **M** and the circumferential direction, and **M**_n =_ **e**_*r*_ represents the unit out-of-plane vector.

Here, *a* and *b* are concentration parameters, $I_{0}(a)=1∕\pi \int_{0}^{\pi }\text{exp}(a\;\cos\;\alpha )d\alpha$ is the modified Bessel function of the first kind of order 0, *α* is the angle between the mean fiber direction and the circumferential direction (see Fig. 3), the in-plane and out-of-plane distributions follow the properties *ρ*_ip_ (Φ) = *ρ*_ip_ (Φ + *π*), *ρ*_op_ (Θ) = *ρ*_op_ (−Θ), and erf is the error function defined as

(5) $$\text{erf}(\sqrt{2b})=\frac{2}{\sqrt{\pi }}\int_{0\;\;}^{\;\;\sqrt{2b}}\text{exp}\;(-\xi^{2})d\xi .$$

Finally, two scalar quantities *κ*_ip_ and *κ*_op_, which measure the in-plane and out-of-plane dispersions, respectively, are defined as

(6) $$\kappa_{\text{ip}}=\frac{1}{2}-\frac{I_{1}(a)}{2I_{0}(a)}\;,$$

(7) $$\kappa_{\text{op}}=\frac{1}{2}-\frac{1}{8b}+\frac{1}{4}\sqrt{\frac{2}{\pi \;b}}\frac{\text{exp}\;(-2b)}{\text{erf}(\sqrt{2b})}\;,$$

where 0 ≤ *κ*_ip_ ≤ 1, 0 ≤ *κ*_op_ ≤ 1/2, and $I_{1}(a)=1∕\pi \int_{0}^{\pi }\text{exp}(a\;\cos\;\alpha )\cos\;\alpha \;d\alpha$ is the modified Bessel function of the first kind of order 1. Larger *κ*_ip_ and *κ*_op_ indicate greater in-plane and lower out-of-plane dispersion, respectively, and in the case of a perfect fiber alignment [36] *κ*_ip_ = 0 and *κ*_op_ = 1/2.

The images were classified as isotropic or anisotropic by fitting a first-order polynomial to the angular distribution of fibers using a threshold value *R*^2^, for more detail see [53]. The value of *R*^2^ = 0.9998 corresponded to the visible fiber morphology and it was chosen as the isotropy threshold. In the isotropic case, a value of 0 was assigned to the concentration parameter, i.e. *a* = 0 or *b* = 0, and no value was assigned to the fiber peak location *α*. In the anisotropic case, the fiber peak locations and the concentration parameters were obtained by fitting the data to *ρ*_ip_ (Φ) or *ρ*_op_ (Θ). Finally, the mean fiber angle *α* of the specimen was calculated by using the standard deviation of all fiber peak locations obtained from fitting the in-plane images in the stack. The in-plane and out-of-plane dispersion parameters *κ*_ip_ and *κ*_op_ for the specimen were then calculated using the mean concentration parameters *a* and *b*.

### 2.4. Constitutive modeling

#### 2.4.1. The two-fiber family model

We model the planar biaxial stress-stretch behavior of SFAs using a strain-energy function that sums the contributions of the ground substance Ψ_g_, and the two families of collagen fibers Ψ_fi_, with the assumption that the mechanical properties of the two families are the same [36]. The total strain energy Ψ is then given by

(8) $$\Psi =\Psi_{g}(C)+\sum_{i={\text{col}}_{1},{\text{col}}_{2}}\Psi_{\text{fi}}(C,H_{i}),$$

where **C** = **F**^T^**F** is the right Cauchy-Green tensor, with the deformation gradient **F** [72], and **H**_col_1__ and **H**_col_2__ are generalized structure tensors that quantify the fiber dispersion. They are given by

(9) $$H_{i}=AI+BM_{i}⊗\;M_{i}+(1-3A-B)M_{n}⊗\;M_{n}\;,\;\;i={\text{col}}_{1},{\text{col}}_{2}\;,$$

where **I** is the identity tensor, the constants *A* = 2*κ*_op_*κ*_ip_ and *B* = 2*κ*_op_ (1 − 2*κ*_ip_) depend on the dispersion parameters, **M**_col_1__, **M**_col_2__ represent the mean in-plane fiber directions, given by

(10) $$M_{{\text{col}}_{1}}=\cos\;\alpha e_{\theta }+\sin\;\alpha e_{z}\;,\;M_{{\text{col}}_{2}}=\cos\;\alpha e_{\theta }-\sin\;\alpha e_{z},$$

and **M**_n_ is a unit vector normal to the plane spanned by the vectors **M**_col_1__ and **M**_col_2__ that represents the radial direction **e**_*r*_ (Fig. 3).

The contribution of the ground substance to the strain energy is assumed to be according to the neo-Hookean model, i.e.

(11) $$\Psi_{g}=\frac{c_{2}}{2}(I_{1}-3)\;,$$

where *I*_1_ = tr**C** is the first invariant of **C**, and *c*_2_ is a material parameter. The strain energy for the two collagen fiber families is considered in the form

(12) $$\Psi_{\text{fi}}=\frac{k_{1}}{2k_{2}}\;\{\text{exp}\;[k_{2}E_{i}^{2}]-1\},\;\;i={\text{col}}_{1},{\text{col}}_{2},$$

where *k*_1_ and *k*_2_ are material parameters and *E*_*i*_ is a Green-Lagrange strain-like quantity, which depends on the fiber dispersion through the structure tensors **H**_*i*_ and the deformation through **C**. By using (9), we have

(13) $$E_{i}=H_{i}\;:(C-I)=AI_{1}+BI_{i}+(1-3A-B)I_{n}-1,\;\;i={\text{col}}_{1},{\text{col}}_{2}\;,$$

where the required invariants read

(14) $$I_{i}=C\;:(M_{i}\;⊗\;M_{i}),\;\;i={\text{col}}_{1},{\text{col}}_{2}\;,\;I_{n}=C\;:(M_{n}\;⊗\;M_{n}).$$

Since *I_i_* is the same for both collagen families we obtain from (12) that

(15) $$\psi_{\;i}^{\prime }=\frac{\partial \Psi_{\text{fi}}}{\partial E_{i}}=k_{1}E_{i}\;\text{exp}\left(k_{2}E_{i}^{2}\right),\;\;i={\text{col}}_{1},{\text{col}}_{2}\;.$$

Hence, with (11), (12) and the strain-like quantity (13) we obtain derivatives of the strain-energy function (8) as

(16) $$\frac{\partial \Psi }{\partial I_{1}}=\frac{c_{2}}{2}+2A\psi_{\;{\text{col}}_{1}}^{\prime },\;\frac{\partial \Psi }{\partial I_{i}}=B\psi_{\;i}^{\prime },\;\;i={\text{col}}_{1},{\text{col}}_{2},\;\;\frac{\partial \Psi }{\partial I_{n}}\;\;=2(1-3A-B)\psi_{\;{\text{col}}_{1}}^{\prime }.$$

For an incompressible material (det**F** ≡ 1) the Cauchy stress tensor ***σ*** can be written as

(17) $$\sigma =-pI+2F\frac{\partial \Psi }{\partial C}F^{T},$$

where *p* is the Lagrange multiplier that enforces incompressibility. Hence, with (16) the non-zero components of the Cauchy stress are

(18) $$\sigma_{\theta \theta }=\left[c_{2}+4(A+B\cos^{2}\alpha )\;\psi_{\;{\text{col}}_{1}}^{\prime }\right]\lambda_{\theta }^{2}-p,$$

(19) $$\sigma_{zz}=\left[c_{2}+4(A+B\sin^{2}\alpha )\;\psi_{\;{\text{col}}_{1}}^{\prime }\right]\lambda_{z}^{2}-p\;,$$

(20) $$\sigma_{rr}=\left[c_{2}+4(1-2A-B)\;\psi_{\;{\text{col}}_{1}}^{\prime }\right]\lambda_{r}^{2}-p.$$

During planar biaxial tests *σ*_*rr*_ = 0 so that (20) can be used to obtain *p*, i.e.

(21) $$p=[c_{2}+4(1-2A-B)\psi_{\;{\text{col}}_{1}}^{\prime }]\lambda_{r}^{2}\;.$$

#### 2.4.2. The four-fiber family model

Previous SFA histological analyses [5,63,64,73] demonstrated longitudinally-oriented elastic fibers in the external elastic lamina and circumferentially-oriented smooth muscle cells (SMCs). To account for these, as an alternative to the two-fiber family model, we consider here two additional fiber families with perfect alignment, i.e., *κ*_ip_ = 0 and *κ*_op_ = 1/2, the longitudinal elastin with *α* = *π*/2, and the circumferential SMCs with *α* = 0. Following the steps described above for the two-fiber family model, one can find *A* = 0, and *B* = 1, for the elastin and SMCs.

The four-fiber family model has a similar strain-energy function as the two-fiber family model, and is given by

(22) $$\Psi =\Psi_{g}(C)+\sum_{i={\text{col}}_{1},{\text{col}}_{2},\;\text{el},\;\text{smc}}\Psi_{\text{fi}}(C,H_{i}),$$

where the ground substance contribution is equivalent to the two-fiber family model, i.e. Ψ_g_ = *c*_4_ (*I*_1_ − 3)/2, with the related material parameter *c*_4_. The structure tensors **H**_*i*_, *i* = col_1_, col_2_ are according to (13), while **H**_el_ and **H**_smc_ are given as

(23) $$H_{i}=M_{i}⊗M_{i}\;\;\;\;i=\text{el},\;\text{smc},$$

where **M**_el_ = **e**_z_ and **M**_smc_ = **e**_*θ*_. For the summation of collagen, elastin and passive SMC, we take a similar expression as in (12), i.e.

(24) $$\sum_{i={\text{col}}_{1},{\text{col}}_{2},\;\text{el},\;\text{smc}}\Psi_{\text{fi}}(C,H_{i})=\sum_{i={\text{col}}_{1},{\text{col}}_{2}}\frac{k_{1}^{\text{col}}}{4k_{2}^{\text{col}}}\left\{\text{exp}\;[k_{2}^{\text{col}}E_{i}^{2}]-1\right\}\;+\frac{k_{1}^{\text{el}}}{4k_{2}^{\text{el}}}\left\{\text{exp}\;[k_{2}^{\text{el}}E_{\text{el}}^{2}]-1\right\}+\frac{k_{1}^{\text{smc}}}{4k_{2}^{\text{smc}}}\left\{\text{exp}\;[k_{2}^{\text{smc}}E_{\text{smc}}^{2}]-1\right\},$$

where *E*_col_1__, *E*_col_2__ are according to (13), while *E*_el_ and *E*_smc_ take on the forms $\lambda_{z}^{2}-1$ and $\lambda_{\theta }^{2}-1$, respectively, and $k_{1}^{\text{col}}$, $k_{2}^{\text{col}}$, $k_{1}^{\text{el}}$, $k_{2}^{\text{el}}$, $k_{1}^{\text{smc}}$, $k_{2}^{\text{smc}}$ are the related constitutive model parameters. Similar to the two-fiber family model, from (17) we now obtain the non-zero components of the Cauchy stress, i.e.

(25) σθθ={c4+2(A+Bcos2α)ψcol1′}+k1smc{(λθ2−1)exp[k2smc(λθ2−1)2]}λθ2−p,

(26) σzz={c4+2(A+Bsin2α)ψcol1′+k1el(λz2−1)}{exp[k2el(λz2−1)2]}λz2−p,

(27) $$\sigma_{rr}=\left[c_{4}+2(1-2A-B)\psi_{\;{\text{col}}_{1}}^{\prime }\;\right]\lambda_{r}^{2}-p,$$

Where again during planar biaxial tests *σ*_*rr*_ = 0. Note that here $\psi_{{\text{col}}_{1}}^{\prime }$ is a modified version of (15)_2_, i.e. $\psi_{{\text{col}}_{1}}^{\prime }=k_{1}^{\text{col}}E_{{\text{col}}_{1}}\;\text{exp}(k_{2}^{\text{col}}E_{{\text{col}}_{1}}^{2})$, where *E*_col_1__ is provided in (13)_2_.

#### 2.4.3. Constitutive parameter determination

The constitutive parameters *c*_2_, *k*_1_, *k*_2_ (for the two-fiber family model) and *c*_4_, $k_{1}^{\text{col}}$, $k_{2}^{\text{col}}$, $k_{1}^{\text{el}}$, $k_{2}^{\text{el}}$, $k_{1}^{\text{smc}}$, $k_{2}^{\text{smc}}$ (for the four-fiber family model) were determined by using the Levenberg–Marquardt algorithm to minimize the error between the theoretical stresses calculated using the equations above and the experimental stresses assessed during the biaxial test, i.e.

(28) $$e=\sum_{i}^{n}{\left(\sigma_{\theta \theta }^{\text{exp},i}-\sigma_{\theta \theta }^{\text{th},i}\right)}^{2}+{\left(\sigma_{zz}^{\text{exp},i}-\sigma_{zz}^{\text{th},i}\right)}^{2}.$$

here *e* is the error function, *i* represents each stress-stretch data point, *n* is the number of all stress-stretch data points that ranged from ~21000 to ~42000 depending on the specimen compliance, while exp and th represent experimental and theoretical stresses, respectively. To ensure parameter uniqueness, a non-parametric bootstrap with 2000 iterations of random sampling and fitting was performed, and the probability distribution of each parameter was analyzed to determine the global minimum [62,74].

The goodness of fit to the experimental data was assessed using the coefficient of determination *R*^2^ calculated as [6]

(29) $$R_{\theta }^{2}=1-\frac{\sum_{i}^{n}\left(\sigma_{\theta \theta }^{\text{exp},i}-\sigma_{\theta \theta }^{\text{th},i}\right)}{\sum_{i}^{n}\left(\sigma_{\theta \theta }^{\text{exp},i}-\sigma_{\theta \theta }^{\text{exp},\;\;\;\text{avg}}\right)},\;\;\;\;R_{z}^{2}=1-\frac{\sum_{i}^{n}\left(\sigma_{zz}^{\text{exp},i}-\sigma_{zz}^{\text{th},i}\right)}{\sum_{i}^{n}\left(\sigma_{zz}^{\text{exp},i}-\sigma_{zz}^{\text{exp},\;\;\;\text{avg}}\right)}\;.$$

Here $\sigma_{\theta \theta }^{\text{exp},\;\;\text{avg}}$ and $\sigma_{zz}^{\text{exp},\;\;\text{avg}}$ are the averages of the experimental stresses, and $R_{\theta }^{\;2}$ and $R_{z}^{2}$ are the coefficients of determination in the circumferential and longitudinal directions, respectively.

### 2.5. Statistical analysis

The Pearson correlation coefficient *r* was used to assess the strength of the linear relationship between the structural parameters and age, with values closer to ±1 demonstrating stronger relations. Statistical significance of the observed correlations was assessed by testing the hypothesis of no correlation (i.e., the null hypothesis) against the alternative hypothesis of nonzero correlation using independent sample *t*-tests. The analyses were performed in the Python programming language. Values of *p*<0.05 were considered statistically significant.

## 3. Results

### 3.1. SFA mechanical characteristics

**Fig. 4.**
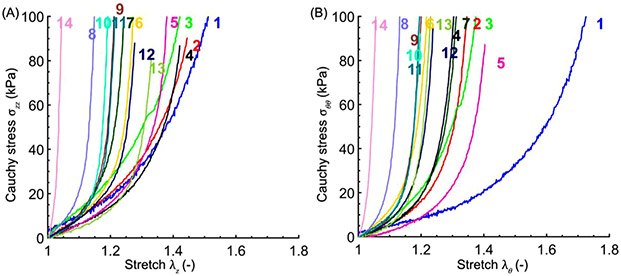
Experimental equibiaxial Cauchy stress-stretch responses in (A) the longitudinal and (B) the circumferential direction for all 14 tested arteries. The numbers on top of the curves are subject numbers (see Table 1).

**Fig. 5.**
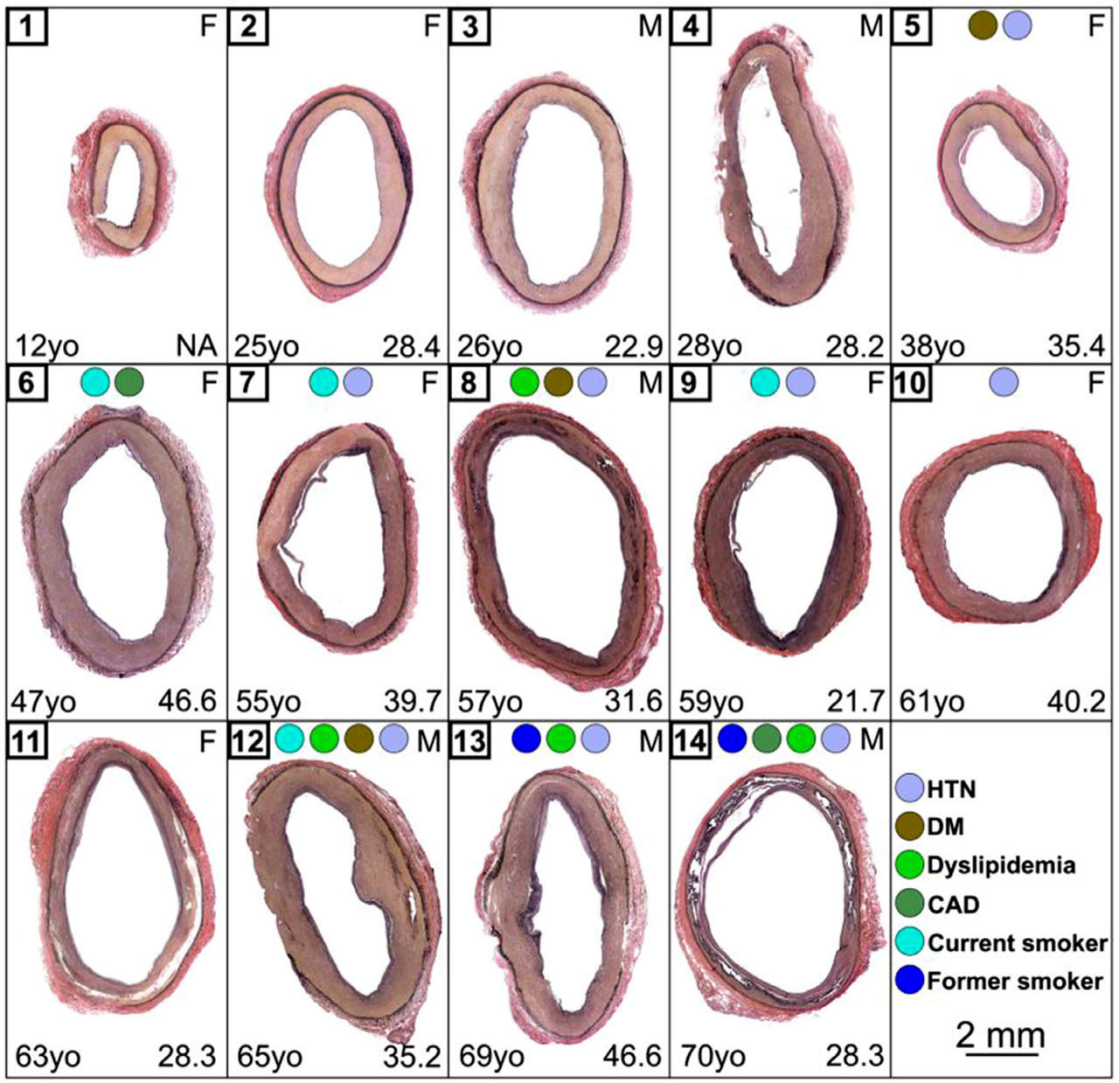
VVG-stained cross-sections for all 14 analyzed arteries. VVG: elastin = black, collagen = red, smooth muscle = brown. Subject number, gender, age, and BMI are reported in the top left, top right, bottom left, and bottom right corners of each box, respectively. Colored circles represent the risk factors for each subject (see the legend on the right). HTN = hypertension, DM = diabetes mellitus, CAD = coronary artery disease.

The equibiaxial stress-stretch curves in the longitudinal and circumferential directions are presented for all 14 tested specimens in Fig. 4(A) and (B), respectively. In general, older SFAs were stiffer, and their stress-stretch responses were more nonlinear with a shorter toe region than those of the younger specimens. However, vascular disease (summarized for each specimen in Table 2 and Fig. 5) also had a considerable stiffening effect, and the more diseased but younger SFAs (i.e., No 8 – a 57-year-old artery) were sometimes stiffer than the older but less diseased arteries (i.e., No 13 – a 69-year-old artery).

### 3.2. Histology

**Fig. 6.**
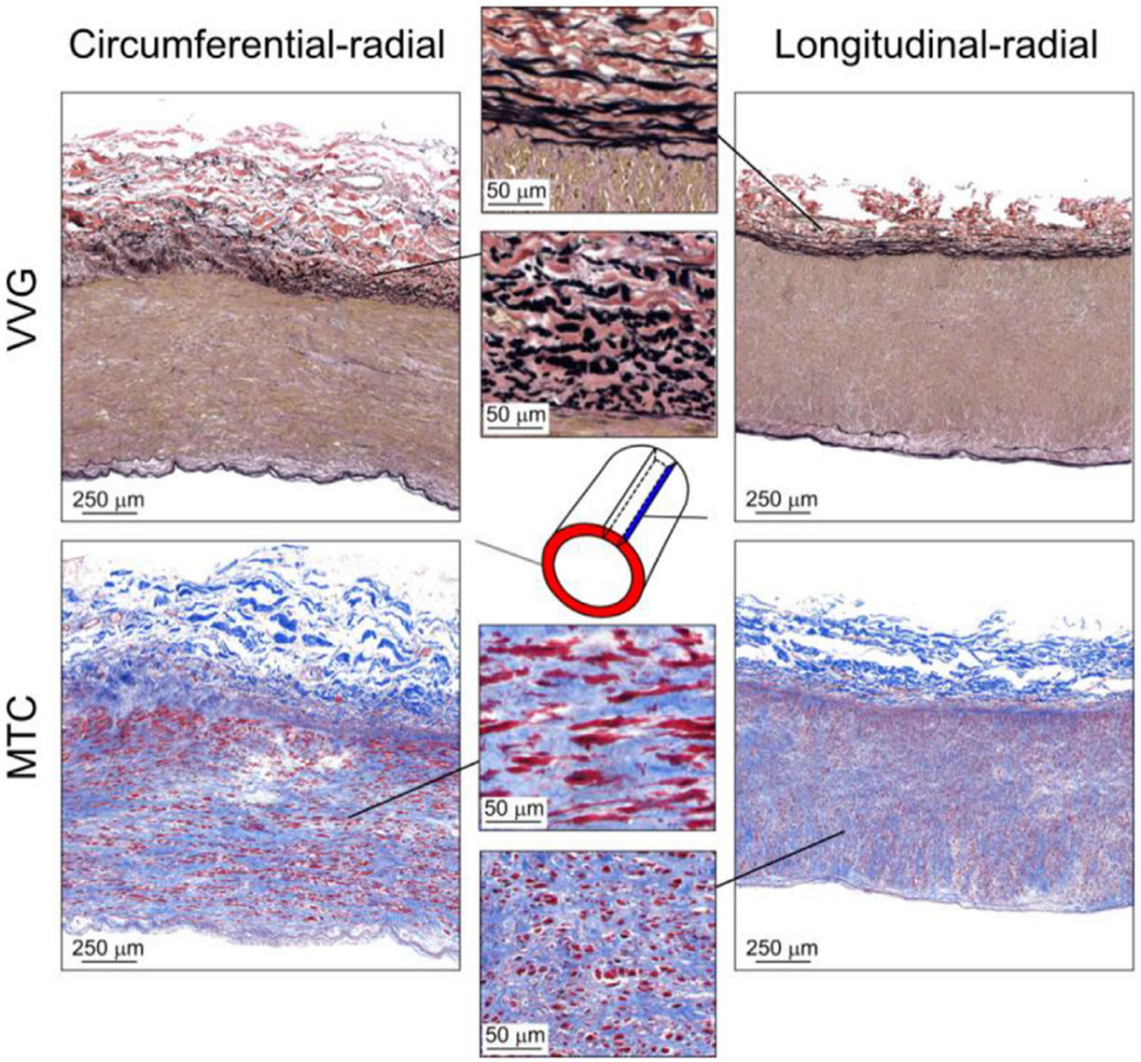
Representative 2D histological images from a 47-year-old SFA stained with Verhoeff-Van Gieson (VVG, top) and Masson’s Trichrome (MTC, bottom) stains. Panels represent the cross-sections (left) and the longitudinal strips (right). Inserts demonstrate that the elastic fibers in the external elastic laminae are oriented longitudinally, and the smooth muscle cells in the media are oriented primarily circumferentially. Collagen surrounds smooth muscle cells in the media, and it is present in the form of undulated fibers in the adventitia. VVG: elastin = black, collagen = red, smooth muscle = brown; MTC: collagen = blue, smooth muscle = red.

VVG-stained cross-sections for all 14 analyzed specimens are presented in Fig. 5 which illustrates changes in the arterial cross-section with age and the heterogeneity of vascular pathology around the circumference. Younger samples (No 1, 2) did not show pathological changes. As the age increased, intimal thickening changed from mild (No 3-6) to moderate (No 7-14), and older samples developed sever medial calcification (No 14). Thick elastic structures were primarily present in the internal and external elastic laminae, with little elastin in the tunica media (Fig. 6). Elastin formed a 3D sheet-like structure in the internal elastic lamina, while it was organized in the form of longitudinal fibers in the external elastic lamina. The MTC stain demonstrated the presence of collagen around primarily circumferentially-oriented SMCs in the media, and undulated collagen fibers in the adventitia.

### 3.3. Multiphoton microscopy

**Fig. 7.**
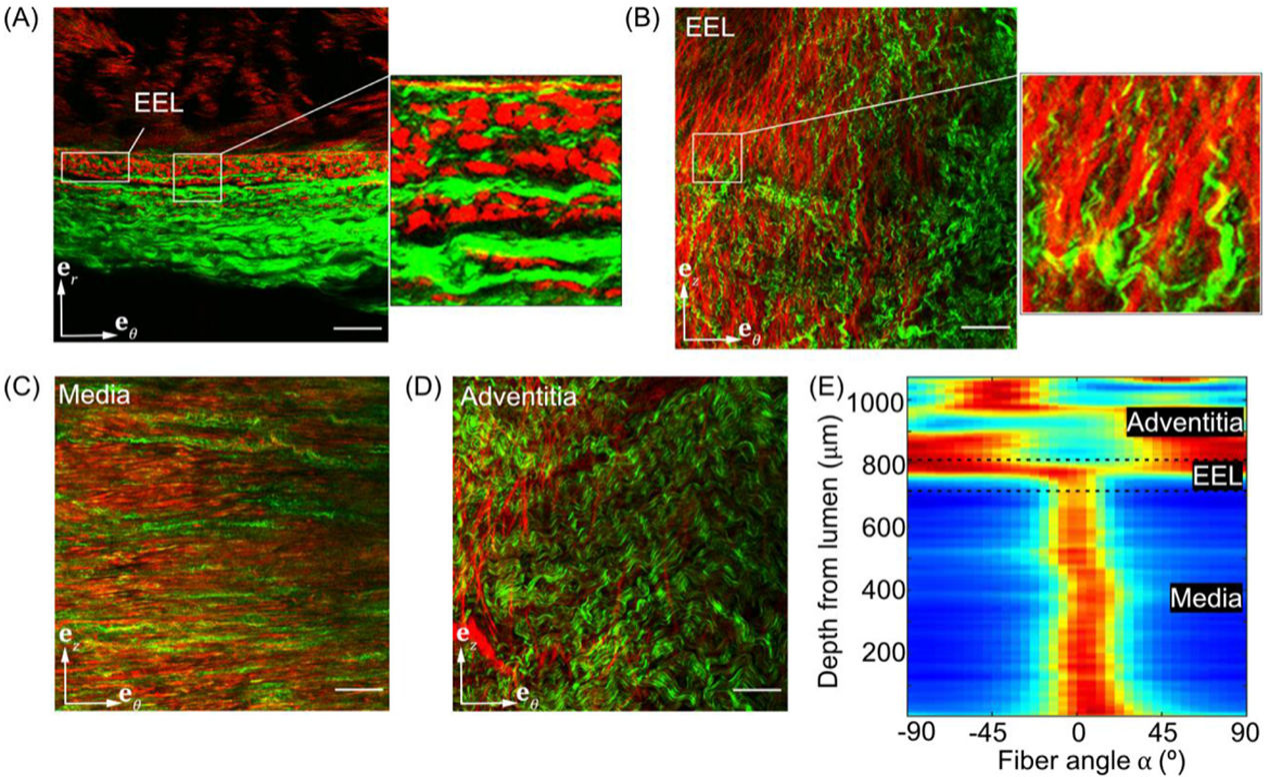
Representative microstructural variation through the thickness of a 47-year-old SFA: (A)–(D) Multiphoton microscopy images with second-harmonic generation (green) and two-photon fluorescence signals (red) showing (A), (B) longitudinally-oriented elastic fibers (red) in the external elastic lamina (EEL) and collagen fibers of type I (green) near the EEL, (C) straight and circumferentially-oriented collagen fibers in the tunica media, and (D) undulated diagonally-oriented collagen fibers (green) in the adventitia. Note the difference in collagen fiber waviness (in green) between (B) and (D). Panel (E) depicts the intensity plot of collagen fibers throughout the arterial wall thickness. Dark red and dark blue colors indicate the presence and absence of collagen fibers, respectively. Scale bar: 100*μ*m.

Fig. 7 illustrates a representative microstructure of the SFA from a middle-aged donor (47-year-old). In agreement with the histological observations (Fig. 6), the elastic fibers in the external elastic lamina were longitudinally-oriented, and appeared very bright (red in panels A and B). The red autofluorescence signal in the medial layer (C) was possibly generated by the autofluorescence of collagen, as the histological images in Fig. 6 demonstrated almost no elastin but a sizable amount of collagen in the tunica media. The medial collagen (both the autofluorescence, red, and the SHG, green, in panel C) were primarily straight and circumferentially-oriented, while the adventitial collagen (green in panel D) was undulated and oriented helically. Collagen fibers of type I (green) were less undulated and appeared thinner in the immediate proximity of the external elastic lamina (B) than the fibers deeper in the adventitial layer (D). The intensity plot (E) shows the variation in the collagen fiber angle *α* through the arterial wall thickness and confirms observations made using panels A-D.

**Fig. 8.**
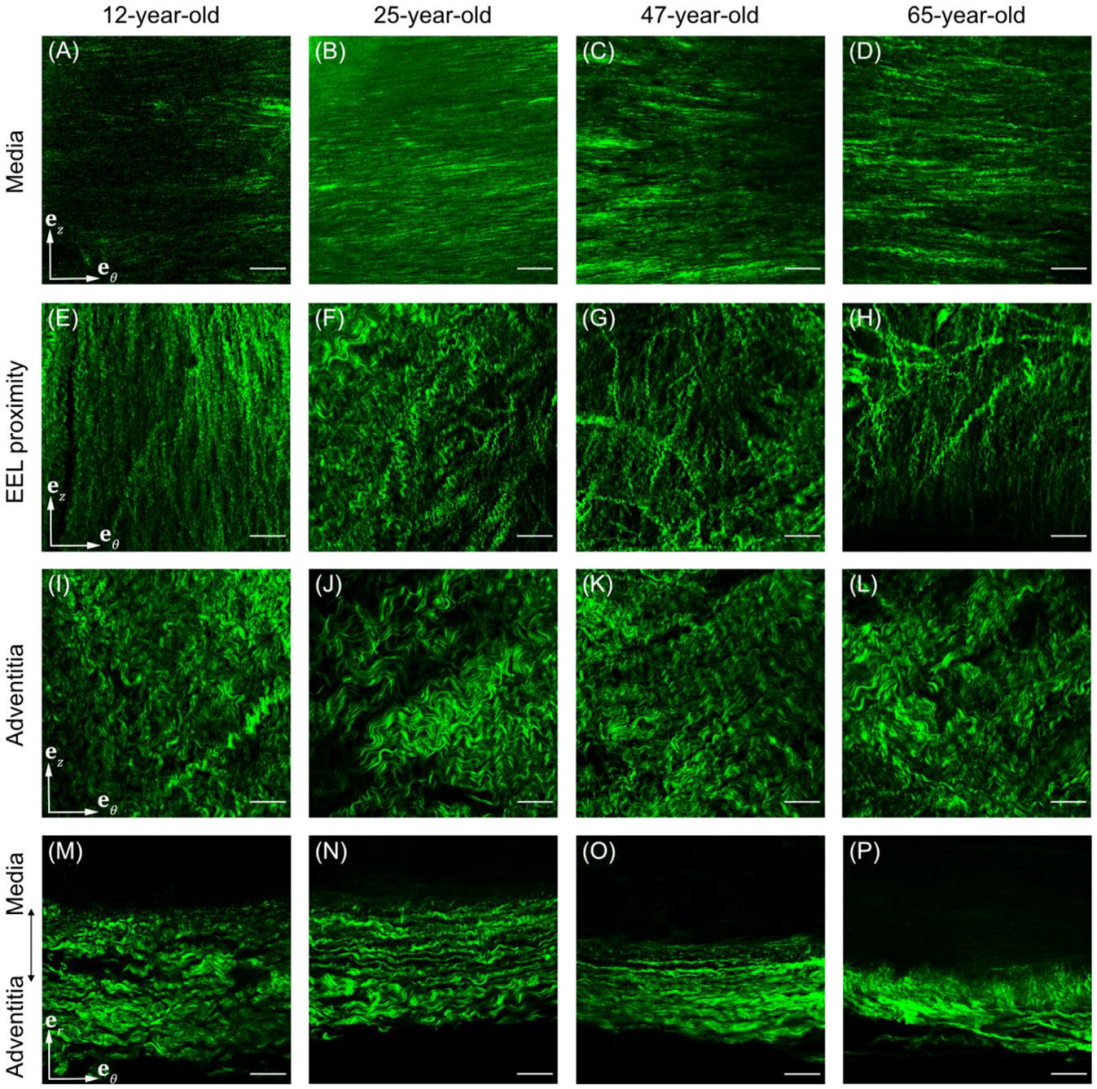
SHG images of a 12-year-old, 25-year-old, 47-year-old, and 65-year-old SFA showing the collagen fiber structure in the circumferential-longitudinal plane of (A)–(D) the tunica media, (E)–(H) the immediate proximity of the external elastic lamina (EEL), and (I)–(L) deeper in the adventitia. Bottom row (M)–(P) illustrates the radial compaction of collagen fibers in the older specimens. Note the different waviness and thickness of the collagen fibers in the immediate proximity of EEL and deeper in the adventitia, as well as the structural differences in the fibrillar collagen with age. Scale bar: 100*μ*m.

Microstructural differences between the SFA specimens of different ages are summarized in Fig. 8. Older arteries had more circumferentially-oriented and thicker-appearing collagen fibers in the tunica media than younger SFAs (A)-(D), and the longitudinally-oriented collagen fibers in the immediate proximity of the external elastic lamina were more disorganized and less wavy (E)-(H). Deeper in the adventitia, collagen fibers of the older SFAs were much less undulated, more circumferentially-aligned (I)-(L), and radially compacted (M)-(P) compared to those in the younger specimens.

**Fig. 9.**
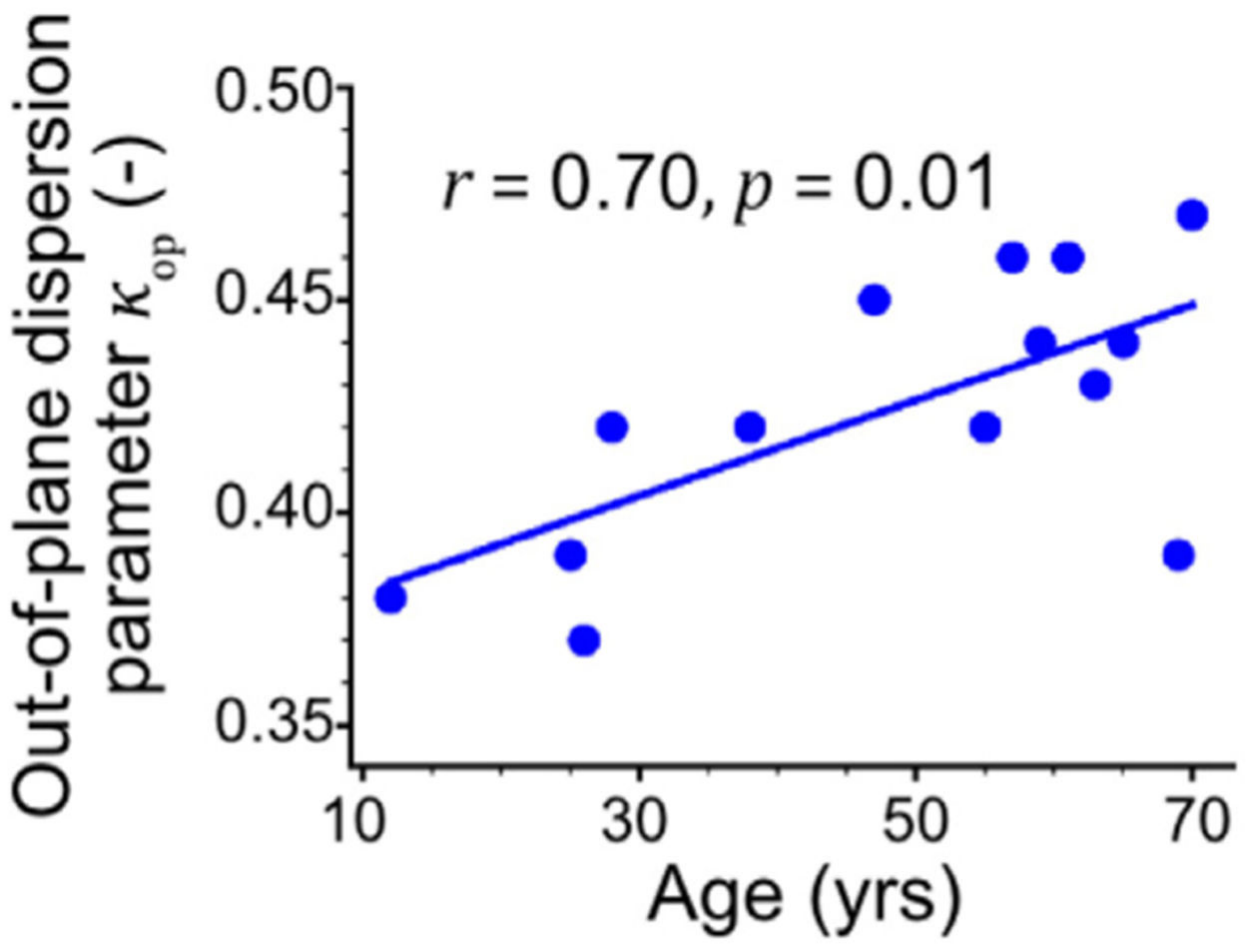
Out-of-plane dispersion parameter *κ*_op_ of all studied SFAs plotted by age. Solid line describes a linear regression between the structural parameter *κ*_op_ and age. Pearson correlation coefficient *r* assesses the strength of this relation, and the *p*-value indicates its significance.

The structural parameters describing the mean collagen fiber angle, and the in-plane and out-of-plane dispersions are summarized in Table 2 and Fig. 9. The mean collagen fiber angle decreased slightly with age (*r*=−0.41, *p*=0.15) from ~ 50° in young to ~ 40° in older subjects, contributing to a more circumferential collagen fiber alignment. The in-plane dispersion parameter *κ*_ip_ remained almost constant with age (*r*=−0.25, *p*=0.40) at ~0.16, and the out-of-plane dispersion parameter *κ*_op_, as shown in Fig. 9, increased significantly with age (*r*=0.70, *p*=0.01, *κ*_op_ = 0.0011 × Age + 0.37) from 0.38 to 0.44, demonstrating less collagen fiber dispersion in the circumferential-radial plane of older specimens.

### 3.4. Constitutive model parameters

**Fig. 10.**
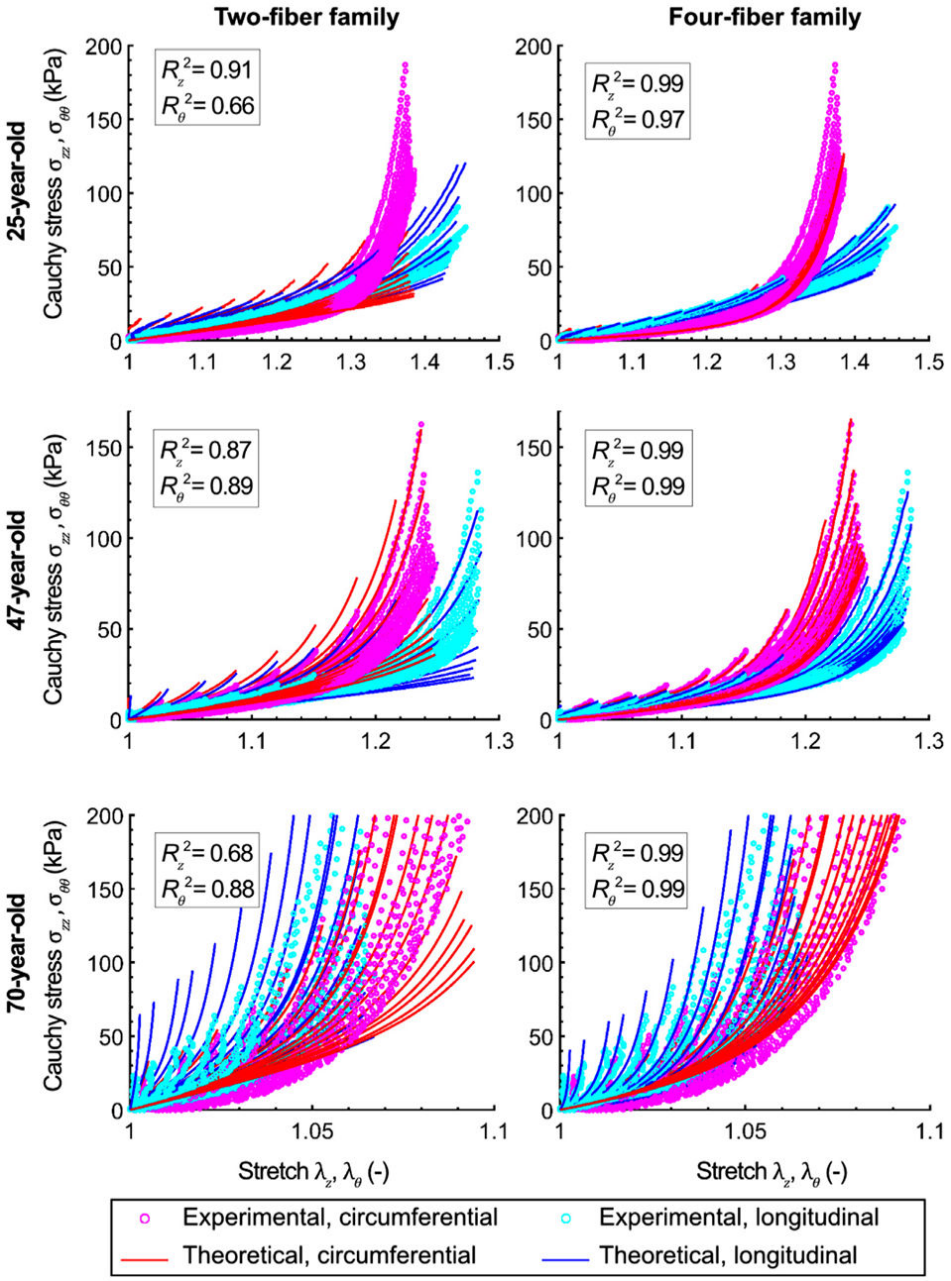
Experimental Cauchy stress-stretch curves from the multi-ratio planar biaxial tests (cyan (longitudinal) and magenta (circumferential) dots), and constitutive model fits (solid blue (longitudinal) and red (circumferential) curves) obtained for the two-fiber family (first column) and the four-fiber family (second column) models. Results presented are for the 25-year-old subject, i.e. #2 (first row), the 47-year-old subject, i.e. #6 (second row), and the 70-year-old subject, i.e. #14 (third row). Coefficients of determination *R*^2^ for the longitudinal direction *z* and the circumferential direction *θ* are also summarized. Please note the different limits of the axes used for better visualization of the fit quality of the older specimens.

Constitutive parameters for the two- and four-fiber family models obtained for each tested specimen are summarized in Table 2. The fits were better when using the four-fiber family model ($R_{z}^{2}=0.98\pm 0.02$, $R_{\theta }^{2}=0.98\pm 0.02$), as opposed to the two-fiber family model ($R_{z}^{2}=0.86\pm 0.09$, $R_{\theta }^{2}=0.81\pm 0.11$). Fig. 10 illustrates these results for three representative samples of different ages.

## 4. Discussion

The ultimate goal of arterial mechanical testing is the determination of constitutive model parameters that allow to simulate arteries *in silico* and assess a variety of mechanical characteristics that cannot be measured *in vivo*. These include intramural stresses occurring as a result of a normal physiologic function or the interaction between the artery and repair devices and materials, residual stresses that are present in the arterial wall, or the elastic strain energy available for pulsation. While it is possible to determine these characteristics using *ex vivo* testing and purely phenomenological models of arterial behavior, more structurally-motivated approaches allow to gain additional insights into arterial physiology and pathophysiology by modeling the function and interaction of various intramural constituents. These constituents primarily include collagen, elastin, smooth muscle cells, and the ground substance containing glycosaminoglycans [13,75], and their distributions and orientations directly influence arterial behavior. Among some of the best-known examples of structural models that explicitly account for arterial intramural components is the constrained mixture theory for growth and remodeling of soft tissues [18,26,76]. This theory provides detailed insights into the tissue function but requires extensive data on each constituent in each configuration to produce reliable results. While many of these parameters are available from well-controlled murine experiments, human data are scarce, necessitating the use of more phenomenological relations to describe arterial mechanics.

Some of the best-known phenomenological models that are used to describe the nonlinear stress-stretch behavior, just capturing isotropy, were proposed by, e.g., Mooney-Rivlin [30,77] and Demiray-Delfino [31,32,78]. While these relations are straightforward to implement in computational analysis, they provide limited insight into the arterial structure and are gradually being displaced by formulations that account for the orientation, dispersion, and undulation of the load-bearing constituents [14,15,34,42,43,45,74,79-81]. The main advantage of these structurally-motivated models is that they can incorporate additional data when available. Developments in advanced imaging modalities allow direct measurements of fiber orientation and dispersion, and these parameters can now be used and included in the constitutive model explicitly instead of being determined through a fitting exercise. Collagen fiber orientation and dispersion have been reported for a variety of vascular tissues [53,55,82], but to our knowledge, this is the first study to report these parameters in human SFAs, which have a distinct structure and function compared with other human arteries [5,6,62,63,83]. They contain longitudinally-oriented elastic fibers in the external elastic lamina at the border of media and adventitia, circumferentially-oriented SMCs in the media, and helically-oriented collagen fibers in the adventitia [63,64,84]. In the present study, we have used SHG combined with TPF to image these features of collagen and elastin and to obtain structural parameters for the two- and four-fiber family invariant-based constitutive models that describe SFA mechanical behavior.

Collagen fibers of types I, III, IV, and V are the most common in arterial walls [13,85]. Among them, only collagen of type I, which is the primary load-bearing collagen [13], gives a strong SHG signal, while all may produce autofluorescence [46,71]. SHG demonstrated a strong signal in the tunica adventitia, similarly to what was reported in other human arteries [53-55]. Adventitial collagen fibers of type I in the SFA were undulated and oriented diagonally, which resembles the organization of adventitial collagen in the healthy human abdominal aorta [50,53]. Older SFAs had straighter adventitial collagen similar to human abdominal aortic aneurysms [50] and elastase treated porcine aortas [86,87], suggesting that collagen straightening may be associated with degradation and fragmentation of elastin [6,62,88] and a shift of the load-carrying capacity to collagen. Similar to reports in other arteries [50,54,89] and non-vascular soft tissues [90,91], our images also showed visibly thicker collagen fibers, which could be attributed to an increase in cross-linking and the accumulation of advanced glycation end-products [92-96], but a more quantitative analysis of collagen fiber thickness in relation to age is warranted.

In addition to adventitial collagen, SHG also demonstrated circumferentially-oriented straight collagen fibers in the tunica media that were more abundant in older SFAs and likely contributed to the increase in their circumferential stiffness and the reduction of the stress-stretch toe region. We have also observed longitudinally-oriented undulated collagen type I fibers in the immediate proximity of the external elastic lamina, and the waviness and thickness of these fibers were different from those deeper in the adventitia. Though the function of these fibers is yet to be determined, their proximity and directional alignment with the prestretched longitudinal elastic fibers in the external elastic lamina likely explain their increased undulation and suggest that they may serve a protective role against axial overstretch that may occur during locomotion.

Changes in the collagen type I fiber architecture with age were revealed by the changes in the in-plane and out-of-plane fiber dispersion parameters. The former remained constant with age, while the latter increased, indicating an increased fiber alignment (i.e., lower dispersion) in the circumferential-radial plane of older specimens. Interestingly, imaging data of the aneurysmal abdominal aorta [50] showed a different trend due to aging with an increase in the in-plane, but a decrease in the out-of-plane dispersion parameters, possibly due to a relatively narrow 53-76-year-old age bracket or a different type of artery.

In addition to imaging collagen type I with SHG, we have also observed collagen autofluorescence in the TPF images of the tunica media. Regarding the SHG images, it is worth noting that the structures in the media visible after contrast enhancement were assumed to be type I collagen, with a weaker SHG signal compared with the adventitia. Although the possible contribution of autofluorescence from other collagen types cannot be excluded, we did not observe a considerable overlap between SHG and TPF images (Fig. 7). Furthermore, histological investigations herein and in the literature [85] suggest that the autofluorescence signal in the TPF images of the media originated likely from type IV collagen surrounding the SMCs. A few MPM studies have discussed the presence of collagen autofluorescence in the media [46,54], but the endogenous autofluorescence emission wavelength for collagen and elastin overlap, which can result in mistakenly attributing this signal to elastin. Separating collagen and elastin signals on MPM is a difficult task [54,97,98], but it can be significantly simplified when supplemented with conventional elastin and collagen-specific histology like it was done here.

The measured structural data informed the constitutive model that was able to capture the characteristic stiffening and increase in nonlinearity in older SFA specimens [5,62]. Though the quality of model fits to the experimental stress-stretch data was similar to the one we reported previously [5,62,73], this new structural information is a step towards more physically-based constitutive formulations that explicitly include fiber angles and dispersions. Perhaps not surprisingly, the four-fiber family model that has more parameters to account for the contribution of the longitudinal elastin and the passive circumferential SMCs in addition to the helically-arranged collagen fibers showed a better representation of the experimental data in specimens of all ages when compared with the two-fiber family model.

While the current study provides valuable information on the microstructure of human SFAs over a wide range of ages and augments its constitutive model with structural data on fiber direction and dispersions, these results need to be also considered in the context of study limitations. First, we have imaged our specimens in the stress-free configuration to inform the constitutive parameters that were also obtained for tissue mechanically stretched from its reference state. Though other studies have performed this imaging in the load-free or loaded states to assess the effects of mechanical load on the microstructure [54,57,86,87,99,100], fiber orientations in other configurations can be determined by applying the appropriate kinematic transformations that will change the reference fiber direction [7,13,14]. Importantly, imaging was performed on non-preconditioned specimens because it required fixing and optical clearing, which would have precluded further mechanical testing. One way to resolve this is to perform biaxial testing and MPM imaging simultaneously, but such a setup was not available to us. Second, while MPM allowed distinguishing layer-specific structural characteristics, the mechanical properties were measured using the arterial wall as a whole, because the atraumatic separation of SFA layers is extremely challenging, particularly in young, healthy specimens. Similarly, MPM allows to image a relatively small segment of the arterial specimen, but the fiber structure may vary significantly both through-the-thickness, as shown in the present study, and along the artery length. Third, while the performed experiments enabled the determination of the collagen fiber direction and dispersion with the goal to incorporate this structural information explicitly into the model, the remaining model parameters are phenomenological, which complicates their comparisons with prior studies of isolated collagen, elastin, or SMCs. While explicitly including structural information into the constitutive relation may not necessarily improve the quality of fits to the stress-stretch data, it does however improve constitutive models and their relevance to particular types of arteries. Such models are then capable of relating the mechanism of deformation to the underlying physical (microscopic) structure of the tissue. Finally, we realize that despite the wide range of ages, our current sample size was quite small and did not allow us to perform meaningful statistical analysis or study the effects of demographics and risk factors on the SFA microstructure. While these limitations are being addressed, our current study informs a more structurally-based constitutive model of SFA behavior that can be used for computational analysis.

## Figures and Tables

**Table 1:** Subject demographics and risk factors.

Subject number	Age	Gender (male/female)	BMI	Never/current/former smoker	HTN	DM	Dyslipidemia	CAD
1	12	F	NA	Never	No	No	No	No
2	25	F	28.4	Never	No	No	No	No
3	26	M	22.9	Never	No	No	No	No
4	28	M	28.2	Never	No	No	No	No
5	38	F	35.4	Never	Yes	Yes	No	No
6	47	F	46.6	Current	No	No	No	Yes
7	55	F	39.7	Current	Yes	No	No	No
8	57	M	31.6	Never	Yes	Yes	Yes	No
9	59	F	21.7	Current	Yes	No	No	No
10	61	F	40.2	Never	Yes	No	No	No
11	63	F	28.3	Never	No	No	No	No
12	65	M	35.2	Current	Yes	Yes	Yes	No
13	69	M	46.6	Former	Yes	No	Yes	No
14	70	M	28.3	Former	Yes	No	Yes	Yes

BMI = body mass index, HTN = hypertension, DM = diabetes mellitus, CAD = coronary artery disease, NA = not available.

**Table 2:** Specimen pathology, structural parameters (mean collagen fiber angle *α* measured from the circumferential direction, in-plane dispersion parameter *κ*_ip_ and out-of-plane dispersion parameter *κ*_op_), and the two- and four-fiber family constitutive parameters determined for each artery.

Subject demographics			Pathology	Collagen fiber angle/dispersion			Two-fiber family parameters					Four-fiber family parameters								
No	Age	M/F		*α* (◦)	*κ*_ip_ (−)	*κ*_op_ (−)	*c*_2_ (kPa)	*k*_1_ (kPa)	*k*_2_ (−)	$R_{\theta }^{2}$	$R_{z}^{2}$	*c*_4_(kPa)	$k_{1}^{\text{el}}(\text{kPa})$	$k_{2}^{\text{el}}\;(-)$	$k_{1}^{\text{smc}}\;(\text{kPa})$	$k_{2}^{\text{smc}}\;(-)$	$k_{1}^{\text{col}}\;(\text{kPa})$	$k_{2}^{\text{col}}(-)$	$R_{\theta }^{2}$	$R_{z}^{2}$
1	12	F	None	61	0.21	0.38	3.22	24.4	0.04	0.95	0.94	7.68	10.63	0.2	0.67	0.52	12.28	0.75	0.99	0.99
2	25	F	None	49	0.12	0.39	15.28	11.59	3.54	0.66	0.91	6.13	10.96	0	2.19	3.96	17.77	2.88	0.99	0.97
3	26	M	Mild intimal thickening	51	0.15	0.37	26.71	13.19	4.17	0.75	0.92	13.46	13.41	0	3.56	2.18	18.12	4.43	0.98	0.97
4	28	M	Mild intimal thickening	43	0.15	0.42	9.82	9.53	3.61	0.73	0.96	3.36	12.06	0	2.79	5.23	9.74	4.29	0.99	0.98
5	38	F	Mild intimal thickening	46	0.17	0.42	4.13	11.98	2.27	0.84	0.83	3.6	9.43	1.12	1.91	1.93	5.11	4.65	0.99	0.98
6	47	F	Mild intimal thickening	36	0.14	0.45	16.72	10.81	10.31	0.89	0.87	13.29	3.79	5.63	6.46	8.04	5.94	14.99	0.99	0.99
7	55	F	Moderate intimal thickening	48	0.14	0.42	0.03	28.68	4.69	0.9	0.74	3.74	18.54	4.24	5.93	2.46	4.3	12.62	0.97	0.93
8	57	M	Moderate intimal thickening, moderate calcification	29	0.16	0.46	25.59	15.61	38.81	0.9	0.81	7.52	17.99	15.47	9.39	26.95	20.2	38.77	0.98	0.99
9	59	F	Moderate intimal thickening	64	0.15	0.44	26.45	1.29	31.93	0.57	0.92	3.28	0.09	0.23	9.63	10.23	13.55	18.71	0.91	0.97
10	61	F	Mild intimal thickening	34	0.16	0.46	20.31	1.52	32.04	0.89	0.73	9.96	4.25	14.7	3.36	11.39	3.84	27.58	0.97	0.98
11	63	F	Moderate intimal thickening, mild calcification, moderate lipid	48	0.18	0.43	10.62	9.85	21.84	0.8	0.88	6.14	11.94	5.64	4.71	11.76	3.44	35.58	0.97	0.96
12	65	M	Moderate intimal thickening, mild calcification	39	0.17	0.44	14.35	4.58	13.17	0.85	0.9	7.26	11.99	2.26	4.34	7.83	3.22	15.41	0.99	0.98
13	69	M	Moderate intimal thickening	42	0.14	0.39	5.81	19.94	12.21	0.78	0.95	2.8	10.07	0.64	5.66	8.57	8.86	21.61	0.99	0.99
14	70	M	Moderate intimal thickening, severe calcification	44	0.13	0.47	119.24	86.65	171.23	0.88	0.68	0	90.99	94.75	189.9	34.46	160.19	162.63	0.96	0.97

## Statement of significance

We have measured the mechanical properties of 14 human superficial femoral arteries using planar biaxial extension, and determined the preferred collagen fiber direction and dispersion using multiphoton microscopy. The mechanical and structural data were used to describe the mechanical behavior using two- and four-fiber family constitutive models. Older femoral arteries were stiffer and mechanically more nonlinear than younger specimens. In the adventitia, collagen fibers were undulated and diagonally-oriented, while in the media, they were straight and circumferentially-oriented. The media was rich in collagen that surrounded the circumferentially-oriented smooth muscle cells, and the elastin was present primarily in the elastic laminae. Both constitutive models were able to capture the experimental data. Our results provide additional details on the intramural structure of superficial femoral arteries and inform structurally-based constitutive models.
